# Surprised by the transition to an unknown body: quantitative and qualitative aspects of activity limitations and physical changes during the first year post-partum

**DOI:** 10.1186/s12884-025-08224-5

**Published:** 2025-10-02

**Authors:** Sabine Vesting, Gun Rembeck, Monika Fagevik Olsén, Annelie Gutke, Maria E.H. Larsson

**Affiliations:** 1 Närhälsan Gibraltar Rehabilitation Centre, Primary Health Care, Gothenburg, Sweden; 2 Department of Health and Rehabilitation, Unit of Physiotherapy, Institute of Neuroscience and Physiology, Sahlgrenska Academy, University of Gothenburg, Gothenburg, Sweden; 3 Research, Education, Development and Innovation, Primary Health Care, Region Västra Götaland, Södra Älvsborg, Sweden; 4 Primary Health Care, School of Public Health and Community Medicine, Institute of Medicine, Sahlgrenska Academy, University of Gothenburg, Gothenburg, Sweden; 5 Regional Health, Youth Guidance Center, Borås, Sweden; 6 Department of Physical Therapy and Occupational Therapy, Sahlgrenska University Hospital, Gothenburg, Sweden; 7 Research, Education, Development and Innovation, Primary Health Care, Region Västra Götaland, Gothenburg, Sweden

**Keywords:** Exercise, Physical activity, Counselling, Pregnancy, Individualized, Pelvic Girdle Pain, Pelvic Floor Muscles

## Abstract

**Background:**

Postpartum women require more individualized support from healthcare providers. However, current care may be insufficient, partly due to a limited understanding of how women experience their symptoms and recovery during this period. The aim was to describe experienced activity limitations during the first year post-partum and to explore and describe women’s experiences of physical changes and recovery after childbirth.

**Method:**

In an observational prospective cohort study, 504 participants reported activity limitations after childbirth via patient-specific functional scales at 3, 6, and 12 months post-partum (*Cohort 1*). The participants also reported causes for these limitations in free-text format. To enrich this dataset with narrative insights, an additional group of 14 women (3─12 months post-partum) was recruited for interviews (*Cohort 2*). The two datasets were analysed via quantitative and qualitative content analysis.

**Results:**

In *Cohort 1*, 48% of the participants reported limitations in high-impact activities (e.g., running, jumping or ball sports) at 3 months post-partum, whereas 41% reported limitations at 12 months post-partum. Other limited activities in the first six months included exercising, lifting/carrying and brisk walks. The main causes for these limitations were pain, vaginal heaviness and urinary incontinence, sensations of instability, and hesitancy to start exercising. *Cohort 2* revealed the theme ‘Surprised by the transition to an unknown body,’ illustrating women’s insecurity about new bodily experiences after childbirth and an experienced need for understanding. The struggle of accepting and adjusting to physical changes contrasts with trust in their bodies’ recovery and ability to adapt life to changes. Recovery can be seen as an accomplishment. Unexpected, unfamiliar physical changes can lead to fear of incomplete recovery and sadness about losing the prepregnancy body. The combination of breastfeeding and hormonal changes while recovering from physical changes can be challenging.

**Conclusion:**

Pain, urinary incontinence, and unfamiliar pelvic sensations, such as vaginal heaviness, can limit postpartum activities. Early limitations seem to arise from uncertainty about feelings of weakness and instability, whereas persistent limitations are usually due to pain or leakage. Early reassurance, information, and tailored guidance could support recovery, while persistent symptoms may require targeted treatment to address evolving needs.

**Supplementary Information:**

The online version contains supplementary material available at 10.1186/s12884-025-08224-5.

## Background

Giving birth is one of the most life-changing events for many women. However, not only the woman’s life but also her body is changing. During pregnancy, the connective tissue of the pelvic floor undergoes structural changes, resulting in lengthening [1], likely due to hormonal influences and increased mechanical load. During childbirth, the pelvic floor muscles must stretch to allow for fetal passage, and following vaginal delivery, these muscles often exhibit reduced strength that can persist for up to a year post-partum [2]. These changes can lead to pelvic floor disorder (PFD), and more than 50% of women experience at least one of the following PFDs: urinary or fecal incontinence, overactive bladder, incomplete defecation or constipation, involuntary flatulence, bulging symptoms and pelvic organ prolapse during the first months after childbirth [3, 4]. Even other muscle groups undergo changes. Owing to the growth of the foetus, the abdominal wall extends during pregnancy, resulting in an increased distance between the two parts of the rectus abdominis during the first year after delivery; this increased distance is called a diastasis recti abdominis [5]. Pain in different parts of the body is common in the postpartum period; 49% of women experience dyspareunia[4], and 33% experience lumbopelvic pain, including pain in both the pelvic girdle and lower back [6]. 

Recent research on postpartum care highlights the imbalance between frequent care during pregnancy and the relative neglect of care in the postpartum period. This phase is marked by women expressing a strong need for support tailored to their individual experiences, including personalized dialogue with healthcare providers and both emotional and physical validation regarding what to expect and how to manage recovery. However, in many countries, the healthcare system does not adequately address these needs [7–9]. In a Swedish survey, 50% of the replying new mothers expressed the need for consultation with a physiotherapist after pregnancy [10]. Physiotherapists work with the evaluation and restoration of physical function on the basis of mutual understanding and agreement with the patient regarding their perceived limitations, their experiences of physical changes and their impact on their daily life [11]. However, currently, physiotherapists are not part of postpartum care in most countries[12], and their knowledge of perceived functional limitations due to the above-described symptoms and how they are experienced by new mothers is limited. Pelvic girdle pain can influence a woman’s ability to take care of the baby and return to prepregnancy activities [13]. Physically active mothers, such as runners, often wish to resume running—a sport that is easily accessible during the postpartum period—approximately 3–4 months after childbirth. However, 30–35% of them report experiencing pain while running [14]. In focus groups discussing postpartum sexual health, women expressed feeling insecure about their physical changes and expressed a need for postpartum check-ups to reassure them that their bodies were returning to normal [15]. Women often feel alone with their physical changes after pregnancy [16]. They want more information about how, for example, pelvic floor tears will recover[17], what changes and symptoms are self-transient and which should be taken care of[18].

Providing individualized support for these physical changes and symptoms remains a challenge for healthcare providers. On the one hand, many postpartum women are in generally good health and capable of performing daily tasks[19], which may lead healthcare providers to overlook their symptoms. Women may self-limit certain activities to avoid discomfort, making their challenges less visible in routine care, where the primary focus is typically on the newborn [7]. On the other hand, expected aspects of muscular recovery—such as soreness, heaviness, or fatigue—can cause significant distress, which may lead some women to overestimate their symptoms or interpret normal recovery processes as problematic. This distress may be amplified by the demands of early motherhood, particularly when essential caregiving tasks such as lifting and carrying become physically challenging, and further compounded by a lack of information, unrealistic expectations, sleep deprivation, and the overwhelming nature of the initial weeks with a newborn [20]. 

Understanding which and why women experience limitations—and why symptoms such as PFDs, low back pain, and pelvic girdle pain contribute to concerns and uncertainty—may be key to improving postpartum care. Therefore, the aim of this study was to describe the most common activity limitations and the causes of these limitations during the first year post-partum and to explore and describe women’s experiences of physical changes and recovery after childbirth to obtain a deeper understanding of their thoughts and concerns about this issue.

We chose a mixed-methods approach to address these aims. Quantitative data from a large cohort identified the types and prevalence of postpartum activity limitations, but many participants also reported a persistent need for support not fully explained by these findings. We therefore conducted a qualitative study to explore the underlying causes, focusing on individuals’ subjective experiences of physical changes and recovery after pregnancy.

## Methods

The first aim was addressed through a secondary analysis of data from the AfterBabyBody Study[21, 22], a longitudinal cohort study conducted between 2018 and 2020. For the second aim, the dataset was enriched with interviews involving 14 additional women conducted between September 2022 and April 2023. Reporting followed standardized checklists: STROBE for observational data and COREQ for qualitative data. Ethical approval for data collection in both cohorts was granted by the Swedish ethical review authority (Dnr 088–18 and 2022–05943–02, respectively). The Declaration of Helsinki was followed, ensuring that all participants had received oral and written information about the studies and given their written approval prior to the start of the study.

### Setting and participants

The first cohort (*n* = 504), also referred to as *Cohort 1*, was recruited by posters at Swedish maternal care and child health care centers in Region Västra Götaland, Sweden, as well as local social media groups and a national blog about women´s health. The inclusion criteria were adults over 18 years of age who had given birth 8–12 weeks prior and who had adequate Swedish language knowledge to fill out the forms. Women with chronic pelvic or back pain, not related to pregnancy, as well as those with major pelvic floor injuries (e.g., obstetric anal sphincter injuries), were not included in the AfterBabyBody Study, as this was an observational study involving repeated pelvic floor and abdominal assessments without offering any treatment, including women with clear therapeutic needs, would have been ethically inappropriate.

The second cohort (*n* = 14), referred to as *Cohort 2*, was recruited at four rehabilitation centers (urban, suburban, and rural) in Region Västra Götaland, Sweden. The physiotherapists at the rehabilitation centers were asked to recruit women who sought help due to concerns about physical changes after pregnancy. The inclusion/exclusion criteria mirrored *Cohort 1*, except for the time after birth, which was 3–12 months post-partum in the interview study. Women interested in participating completed a brief questionnaire with background variables prior to enrollment. Individuals with different background variables (socioeconomic status, education, number of children, time after delivery, and physical activity level) were selected for a variety of data. After four months of recruitment, only participants with a university degree had agreed to participate in the study. An extended recruitment strategy was applied, using similar local social media groups and a national blog about women´s health, as in *Cohort 1*. Five additional participants were found—two with no university degree.

#### Data collection

The data for *Cohort 1* were collected via questionnaires administered at 3, 6, and 12 months post-partum. In the questionnaires, the participants completed a patient-specific functional scale (PSFS) [23]. In our study, the participants could choose up to three activities that they were unable to perform or found difficult due to physical changes related to their pregnancy/delivery (see Appendix). They rated their difficulties from 0 to 10 and provided causes for the activity limitations in free text. Activities rated ≤ 8 on the PSFS were defined as limited. For *Cohort 2*, the data were obtained through semistructured individual interviews. In both cohorts, background data such as age, BMI, educational level, number of children, and delivery mode were collected.

### Semistructured individual interviews

The first author (SV) contacted the eligible participants to book an appointment for an interview. SV had no knowledge of their medical history other than the collected background data. The participants were informed that SV was a PhD candidate but received no further information about her. The place and time for the interview were chosen by the participants. Two participants chose to conduct the interviews in a meeting room at the University of Gothenburg, while the remaining participants found it most feasible to be interviewed by phone. All the participants confirmed that they were in a private setting without disturbances (aside from the presence of their baby) and felt comfortable discussing intimate topics.

All the interviews started with the following question: “How did you experience your body after pregnancy? The further course of the interview was guided by a basic but not rigid interview guide (see *Appendix*), addressing topics such as physical recovery and the impact of changes in physical activity. The interviewer used questions such as “Could you tell us more about your experience? What do you mean?” to delve deeper into participants’ experiences. The questions concerning concerns and uncertainty about physical changes were not asked unless the participants introduced the topic themselves. This approach was intentional, as we aimed to avoid influencing participants’ responses by introducing emotionally charged or ambiguous terms. In this context, concern refers to a feeling of worry or perceived risk, whereas uncertainty denotes a lack of clarity or predictability about a situation. Directly asking about these states could have led participants to reflect on and potentially construct causes for such feelings, even if they were not initially present. However, when such topics were raised spontaneously, they were explored in greater depth. Only the participant, often the participant´s baby, and the interviewer (SV) were present at the interviews. The first interview was conducted as a pilot interview and was discussed by the research group before the other 13 interviews were conducted; however, no significant changes were made, and the interview was included in the analysis. The interviews averaged 39 minutes (ranging from 25–44 minutes), with one participant contributing supplementary information (3 minutes) after the interview.

The interviews were recorded using two methods: the recording function within PowerPoint, installed on a secured server, and an external audio recorder placed next to the computer. This dual setup ensured sound quality and served as a backup in the case of technical issues. The PowerPoint files were saved in a password-protected folder on the interviewer’s computer, accessible only to her and her supervisor. After each interview, the audio files from the external recorder were transferred to the same secure folder and deleted from the recording device to ensure data protection.

#### Data analysis

Microsoft Excel was used for sorting the data of *Cohort 1*, and NVivo was used for sorting the data of *Cohort 2*. The data from the PSFS questionnaires were analysed via quantitative content analysis [24]. All reported limited activities, rated between 0 and 8 (PSFS scale 0–10), were coded, sorted into subcategories and then categorized into comprehensive terms, such as high impact for running, jumping and ball games; childcare for lifting and carrying the baby; and changing diapers (see Table 2). The participants’ causes for activity limitations were analysed following the same steps.

**Fig. 1:**
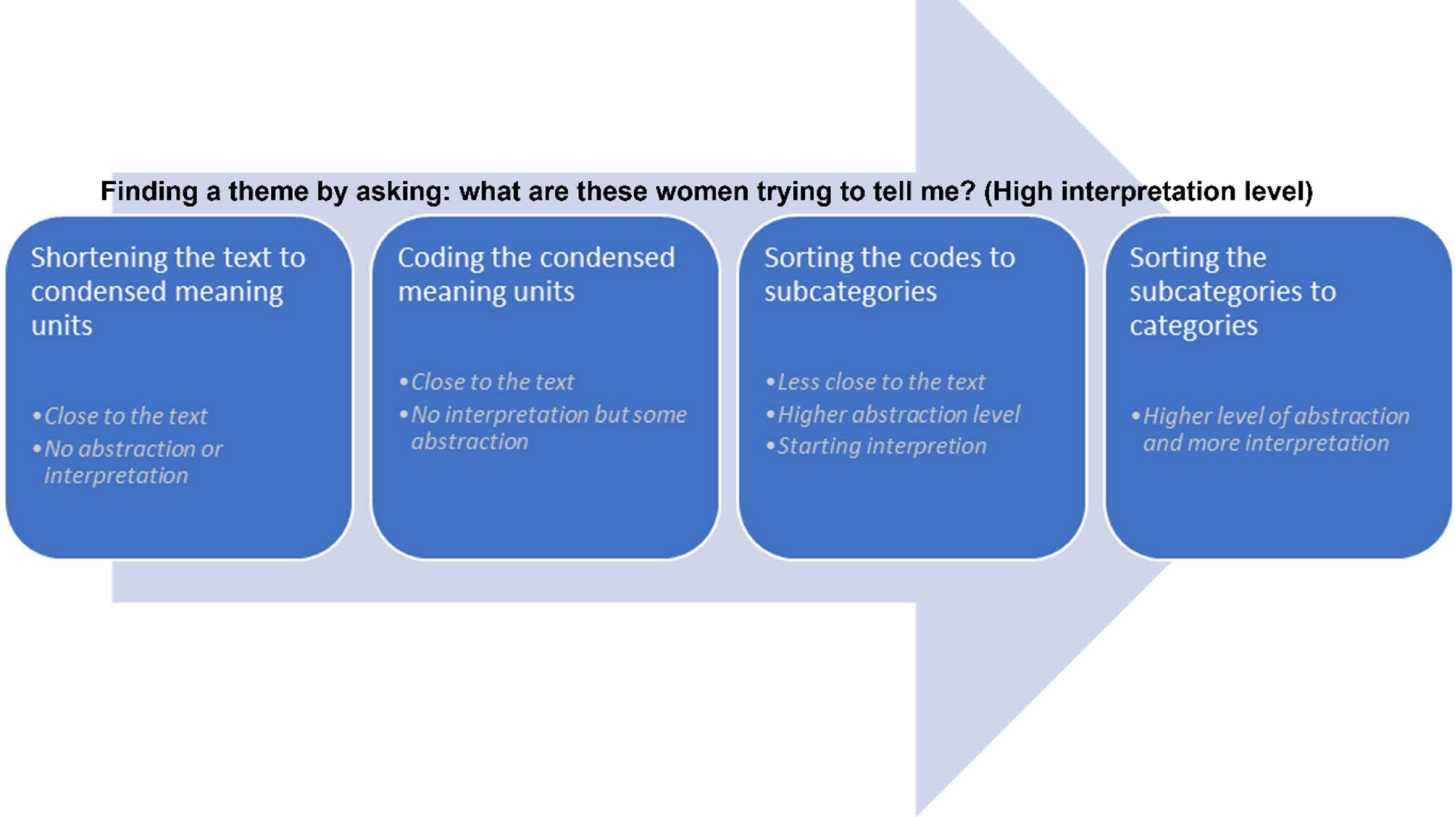
The qualitative content analysis process was performed according to Graneheim and Lundman (reference 25)

The qualitative data were transcribed verbatim and listened to and read several times to grasp the overall content of the texts. Qualitative content analysis, as described by Graneheim and Lundman, was used to analyse the texts [25]. In this analysis process, the researchers followed several steps to understand both the manifest and latent content of the units of analysis, which in this study consisted of interview texts— that is, the entire body of text selected for analysis and used as the basis from which meaning units were identified. The steps are visualized in Fig. 1.

As described in Fig. 1, the researchers followed a congruent pattern to achieve abstraction and interpretation without losing the content of the participant´s experience by starting close to the text and progressively advancing toward greater abstraction and interpretation in the following steps. During this process, the researchers were looking for an underlying meaning or a red thread between the lines to a theme.

The analysis was conducted by two of the researchers (SV, MEHL), who independently condensed, coded and categorized the texts and then discussed them to reach agreement. The initial findings were shared with the entire research team, which prompted further discussion and refinement until a conclusive result was achieved.

No power analysis was conducted for *Cohort 1* because it was a secondary analysis, and no p values are presented. The number of interviews conducted for *Cohort 2* was guided by Malterud’s model for information power [26]. The initial assumption was that a larger sample size was needed due to the use of a broad research question and an inductive approach without an underlying theory. However, the interviews yielded strong-quality dialogues, and through purposive recruitment, participants with diverse experiences related to physical changes and recovery, both with and without underlying concerns, were identified. Moreover, the use of an analysis method that addresses both manifest and latent content further supported the adequacy of a smaller sample size in this study [26]. 

### Researcher characteristics and reflexivity

The first author (SV), a female PhD candidate and clinical physiotherapist (with a focus on postpartum health problems), was primarily responsible for the data collection and analysis. Researcher AG, an associate professor, is a specialized physiotherapist in gynecology, obstetrics, and urology who also works clinically with postpartum patients. The remaining research groups included MEHL, an associate professor and a primary care specialist physiotherapist; MFO, a full professor; a specialist-physiotherapist in gynecology, obstetrics, and urology; and GR, PhD, a registered nurse and registered midwife, all with extensive hospital and primary care experience. In recent years, they have had less contact with the patient group described. All the researchers, except of the PhD candidate, have long and broad experience in qualitative research and closely supervise data collection and analysis. All the authors had their own experience with pregnancy, childbirth, and recovery.

## Results

The results are based on data collected from two cohorts: Cohort 1 (*n* = 504) and Cohort 2 (*n* = 14). The detailed characteristics of both cohorts are presented in Table 1.

**Table 1:** Descriptive presentation of the two study cohorts

			Cohort 1 (*n* = 504)	Cohort 2 (*n* = 14)
Age, mean (SD), range			33.1 (3.6) 24–46	33.6 (4.0) 27–40
BMI, mean (SD), range			24.4 (3.3) 17–37	25.5 (5.8) 20–44
Education level, n (%)		Primary school, high school, or other	46 (9.1)	2 (14.3)
		University or college, less than 3 years	46 (9.1)	0
		University or college, 3 years or longer	410 (81.3)	12 (85.7)
Delivery mode, n (%)		Vaginal delivery	438 (86.9)	12 (85.7)
		Cesarean section	64 (12.7)	2 (14.3)
Pelvic floor tear, n (%)		No tear	96 (19.0)	1 (7.1)
		1 st degree tear	130 (25.8)	2 (14.3)
		2nd degree tear	195 (38.7)	9 (64.3)
Parity, n (%)	Primipara		315 (62.5)	8 (57.1)
	Multipara		187 (37.1)	6 (42.9)
Time after delivery (in months), range			Baseline characteristics measured at 3 months post-partum	6.9 (2.6) 3–12

*BMI* Body mass index, *n* Number, *SD* Standard deviation

### Cohort 1

At 3 months post-partum, 297 out of 504 participants (58.9%) from Cohort 1 reported experiencing activity limitations due to physical changes after pregnancy. Among them, 213 participants reported multiple activity limitations, resulting in a total of 600 reported activities. Six months post-partum, with a dropout rate of 6.3%, 266 participants (56.4%) out of 472 reported a total of 514 limited activities. For the corresponding figures at 12 months post-partum, 186 (43.9%) of the 424 participants had problems with 344 activities. Many of the reported activities were similar or overlapping, as participants used interchangeable terms such as “go for a run”, “jogging”, or “run”. The specific activities are shown in Table 2.

**Table 2:** Perceived activity limitations at 3, 6 and 12 months post-partum

Codes	Subcategories	Categories	3 months post-partum	6 months post-partum*	12 months post-partum
			(*n* = 504)	(*n* = 472)	(*n* = 424)
Jogging	Running	High-impact activities	244 (48.4%)	211 (44.7%)	172 (40.6%)
Running downhills/uphill					
Running to the bus					
Long distance running					
Playing with the older child					
Jumping exercises like jumping jacks, scissor jumps	Jumping				
Trampoline jumping with the older child					
Skipping					
Aerobics/CrossFit					
Volleyball	Ball sports				
Basketball					
Innebandy/squash					
Football					
Abdominal exercises (sit-ups, side and front planks)	Specific exercises	Exercising	105 (20.8%)	78 (16.5%)	49 (11.6%)
Squats					
Lunges					
Yoga					
Skiing/Ice skating	Aerobic training				
Cycling/spinning					
Horse-riding					
Weightlifting	Heavier training				
Strength training					
Climbing					
Lifting the child from the floor	Lifting	Lifting/carrying	64 (12.7%)	50 (10.6%)	22 (5.2%)
Lifting the stroller/parts of the stroller/baby car seat					
Lifting heavy	Lifting/carrying heavy				
Lifting/carrying the older child					
Carrying heavy					
Walking with a stroller	Daily walking	Walking	53 (10.5%)	30 (6.4%)	14 (3.3%)
Walk uphill/downhills					
Longer walks (> 3-10k)	More intense walking				
Power walk					
Hiking					
Carrying the baby	Lifting/carrying the baby	Childcare	26 (5.2%)	26 (5.5%)	20 (4.7%)
Lifting the baby					
Changing diapers	Daily activities with the baby				
Putting the child to sleep					
Playing with the baby on the floor					
Sitting for a longer period (> 10–60 min)	Sitting	Maintaining body positions	23 (4.6%)	16 (3.4%)	12 (2.8%)
Sitting without back support					
Sitting in squat position/cross-legged					
Car-driving					
Standing straight	Standing				
Standing for a longer period of time, i.e., cooking food in standing position					
Standing up from the couch/bed	Standing up from low height	Changing body position	22 (4.4%)	16 (3.4%)	2 (0.5%)
Standing up from the floor					
Standing up with weight from the baby	Using abdominal strength to get up				
Using abdominal muscles to come up					
Bending forward	Leg and back movement	Basic movements	21 (4.2%)	30 (6.4%)	18 (4.2%)
Side steps/pulling the foot inwards					
Crossing legs					
Quick turns/movements	Balance/stability impairments				
Standing on one leg					
Turning over in the bed					
Digging in the garden	Gardening	House-hold tasks	17 (3.4%)	7 (1.5%)	3 (0.1%)
Weeding					
Hanging laundry	Household				
Cooking/washing					
Vacuum cleaning					
Vaginal/penetrating intercourse	Sexual intercourse	Intimacy	15 (3.0%)	26 (5.5%)	11 (2.6%)
Sexual contact					
Positions with the legs apart					
Using a tampon	Inserting aids				
Using a p-ring (contraception)					
Laying on the side	Resting	Resting positions	6 (1.2%)	9 (2.0%)	7 (1.7%)
Laying with straight legs					
Sleeping in a comfortable position	Sleeping				
Sleeping on the back/belly					
Emptying the bladder	Toilet functions	Basic function	4 (0.8%)	8 (1.7%)	11 (2.6%)
Emptying the bowel					
Coughing/sneezing without leaking	Spontaneous functions				
Laughing without leaking					

*n* Numbers

* Five activities were not categorized due to their specificity and lack of alignment with existing categories. Examples include rotational movements with the hands positioned far from the body, and instances where water entered the vagina during bathing or certain yoga poses, resulting in vaginal flatulence

**Fig. 2:**
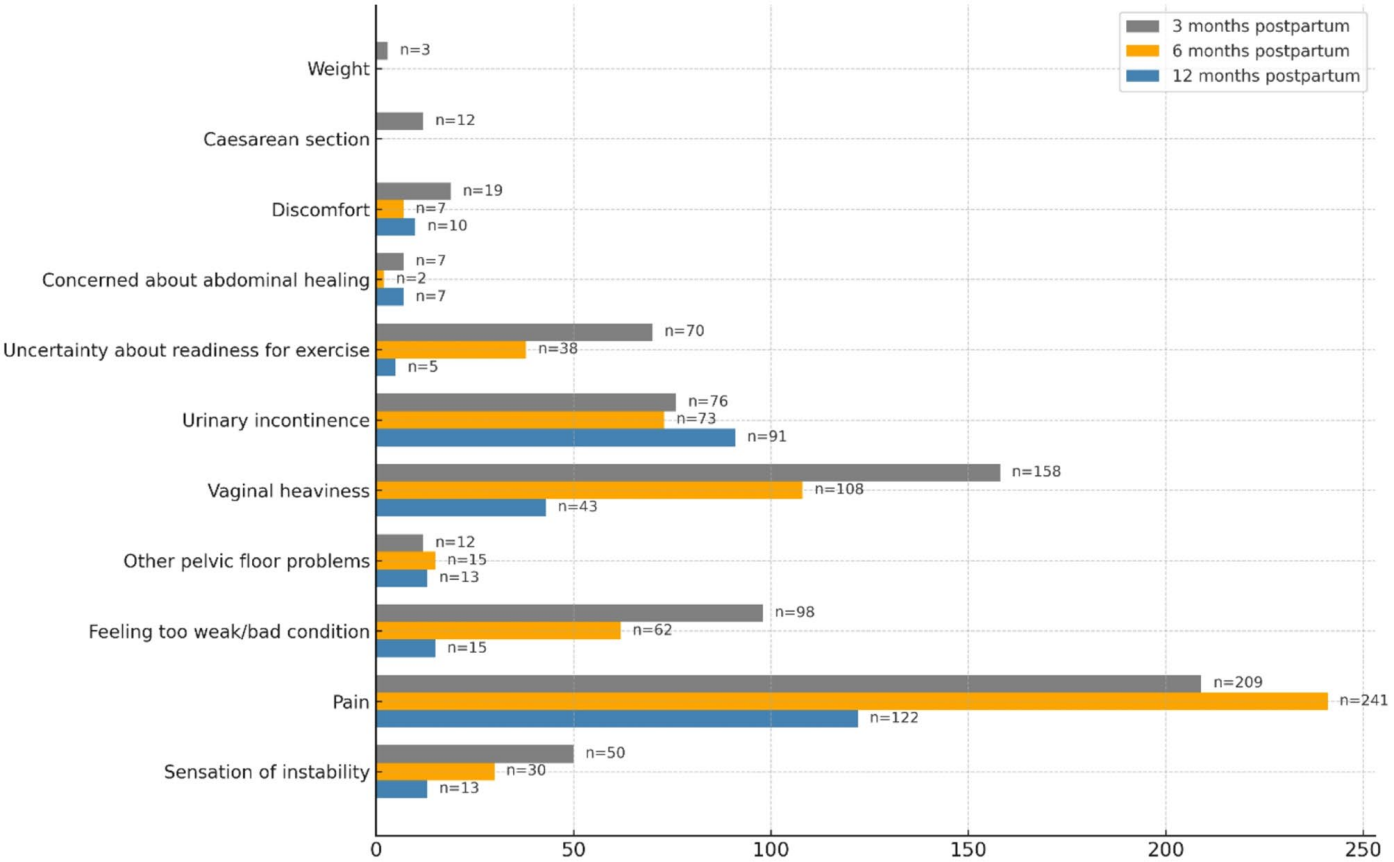
Reported causes of activity limitation at 3, 6, and 12 months postpartum (*Cohort 1*). Bars show the number of participants reporting each concern, with values (n=) indicating the exact number per cause, n, numbers

The main cause of activity limitations was pain (in the pelvis, back, pelvic floor, abdomen, groin, foot, hand joint or knee), which was reported by 34.8%, 46.9%, and 35.5% of participants at 3, 6, and 12 months post-partum, respectively. This pain varies in type, including dull, stabbing, cramping, soreness, and stiffness. Pelvic floor issues are another common cause of activity limitations. Vaginal heaviness limited 26.3%, 21.0%, and 12.5% of activities at 3, 6, and 12 months post-partum, respectively, whereas urinary incontinence was reported as a limitation for 12.7%, 14.2%, and 26.5% of activities at the same time points, respectively. Additional causes are shown in Fig. 2.

### Cohort 2

**Fig. 3:**
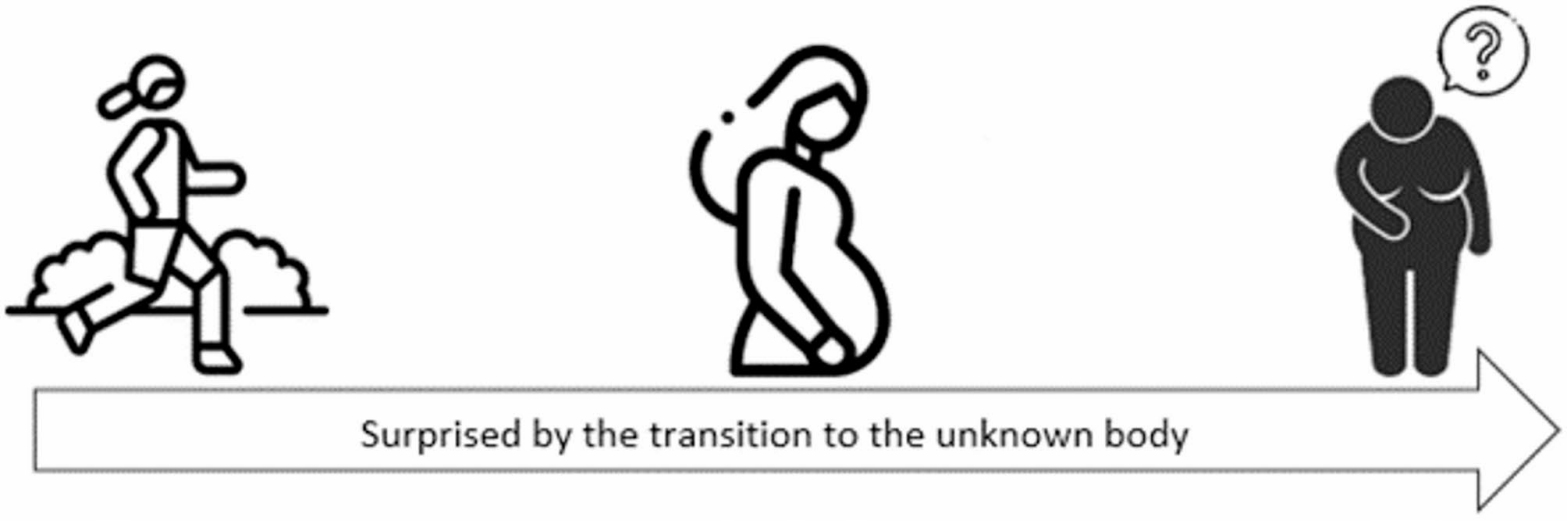
The surprising transition to physical changes and recovery in the postpartum period

The overarching theme that emerged after the analysis of the 14 interview texts was “Surprised by the transition to an unknown body” (Fig. 3), which is the red thread through the categories, subcategories and codes.

Without knowing what is right or wrong, women adjust to changes in function and appearance after childbirth that they often did not anticipate. They search the internet to find explanations and knowledge. The qualitative content analysis of the 14 interviews revealed 7 categories, each with 2 to 5 subcategories, as shown in Table 3. The meanings of the individual categories are described below in detail.

**Table 3:** Overview of the themes, categories, subcategories, and codes that emerged as a result of qualitative content analysis (Cohort 2)

Theme	Categories	Subcategories	Codes
Surprised by the transition to a new body	An unfamiliar bodily sensation	Preparation helps	Having experiences
			Never felt this before
			Being prepared
		Cannot do it alone, help me	Support of family and friends
			Support of health care
			Needing reassurance
			Want to know/understand
			Asking for the new normal
		Taking an active role in recovery	Should I do something
			Needing the right tools
			Needing the right help
	Trusting the body´s ability to recover	Feeling good now	Feeling recovered
			Confident about the body´s recovery
		Changed but acceptable	The body´s function is important
		Getting better and better	Needing rest and time for recovery
			Experiencing small successes
		Having control	Will get strong again
			Being responsible
	Challenge due to the overload of being a mother to a newborn	Feeling limited in energy	Hard to take care about my own recovery
			Stressed by the need to take care of myself
			Need of help
		Overwhelmed of the first weeks	Adding up with other problems
			Pain is secondary due to other problems
			Getting worse by sleep deprivation
		Not as it was expected	More problems than expected
			Wrong expectations
	Recovery as an accomplishment	Disappointed with my body	Pressured by others
			Pressured by myself
			Striving after to be a good mother
		Proud and thankful to the body	Impressed by the body
			Fascinated by the body`s ability to be strong
			Humbled of the body´s abilities
		Comparing to others	Being the only one
			Could be worse
			Terrified by stories of others
	Sadness over losing my prepregnant body	Longing for my prepregnant body	Impatience to live a normal life again
			Focus shifts from mother to baby
			Cannot be as active as I want to be
		Looking normal but feeling different	Feeling/being teared
			Feeling misunderstood
	Struggling with adjustment to physical changes	Experiencing limitations	Experiencing pain/leakage
			Threatening basic function (bowel and bladder)
			Affecting the core
		It´s okay but not forever	Recovery takes time
			Shifting priorities
		A process of accepting	Need to accept changes in appearance
			Need to accept changes in function
		Need of adapting	Adapting my everyday life
			Avoiding activities
		Feeling powerless	Cannot influence my recovery
			Nobody is helping me
	Fear of not regaining my bodily functions	Fear caused by knowledge	Reinforced fear by the internet
			Having risk factors in family history
		Destroyed my body	Unchangeable physical changes
			Scared to be teared
		Needing a strong body to take care of my kids	Need a strong core
			Wondering what´s next
		Afraid of doing something wrong	Might be dangerous
			Feeling unstable

#### An unfamiliar new bodily sensation

This category describes how women experienced physical changes after pregnancy that were unfamiliar to them, something they had never experienced before. They described sensations such as vaginal heaviness, feeling as if their organs lacked support and could fall. Even participants with multiple children expressed new unfamiliar sensations after the recent baby, indicating that their bodies changed with each baby. The subcategory *preparation helps* shows that a lack of prior experience made it challenging to discern whether these new bodily sensations were normal. Women with childbirth experience found comfort in knowing that these sensations were typically temporary. The first days after childbirth were expected to be tough but often not the first weeks or months. There were ambivalent opinions regarding whether prenatal information would be helpful; it could both be unnecessarily scaring or helpfully preparing.

*“Because I have never done anything like this before*,* I did not truly know either; maybe it was supposed to hurt that much.” (Interview 3)*.

The subcategory *Cannot do it alone*,* help me* describes the wish for more help and support during the first months and the possibility of talking about physical changes and recovery and valuing input from mothers, sisters, and healthcare providers. Reassurances or follow-ups from professionals were also requested to confirm the normalcy of symptoms and understand recovery timelines. While hesitant to burden the healthcare system, women still believed in their right to seek help. *Taking an active role* describes women’s need for comprehension; they seek information online to understand their physical changes and recovery trajectory. Support from professionals, such as midwives or physiotherapists, combined with access to practical tools for managing problems, contributed to a sense of security. They expressed a need for clearer guidance regarding expected bodily responses during physical exertion and increased load.

*“(At the physiotherapist)*,* I got concrete help and tools to get better instead of waiting for getting better*,* and then I could talk about my concerns and those things… will this be fine or is it something I have to live with… and I got it (answers)*,* which was truly calming”. (Interview 5)*

#### Trusting the body’s ability to recover

This category reflects participants’ trust in their body’s postpartum recovery. While some no longer experienced changes, others noted gradual improvements. Some found their changed bodies acceptable, as they managed by allowing time for rest and recovery. This is demonstrated in the subcategory of *feeling good now*, where the body was experienced as recovering, strong, and prepared for life with small children, anticipating the return of all its functions over the coming months. Once the symptoms disappeared, they were soon forgotten. Another subcategory, *changed but acceptable*, described how some physical changes could still be present, such as feeling weaker or wider. However, these changes were experienced as acceptable as part of becoming a mother. The postpartum period was seen as a time not focused on physical challenges and exercising, and the women were content with that. They also noted that they were able to make slight adjustments to their daily routines without perceiving them as limitations.

*“I probably would not go on a very long hike at the moment*,* but it is not something that I feel is a problem.” (Interview 2)*.

Furthermore, *having control* indicates that trust in the body is also based on the ability to control physical changes. For example, the need for rest and recovery time, as well as the effects of physical activity, training, and specific exercises, was emphasized. Additionally, the experience of *getting better and better* highlighted small gains, such as taking longer walks, which helped women overcome the perception that these new body sensations were permanent. Postpartum recovery and physical changes were seen as a process, and as long as this process progressed, the participants trusted their body’s recovery.

#### Challenge due to the overload of being a mother to a newborn

This category explores how the demands of motherhood with a newborn affect the experiences of physical changes and recovery after pregnancy. In the subcategory *feeling limited in energy*, the inner conflict was expressed as the trade-off between exercising for long-term energy benefits and the short-term cost of mental and physical energy. Although exercise is viewed as a potential energy source for life with small children, it is not always prioritized.

*Overwhelmed by the first weeks* described the challenges of the initial postpartum period, where the focus was primarily on basic needs and breastfeeding. While some experienced much pain that accumulated with the experience of physical changes, others had almost no time to think about their recovery during the first weeks.

*“During the first few weeks*,* it was about processing it emotionally (cries a little bit)*,* so the body came second. However*,* yes*,* especially the first week*,* I had a lot of pain even though I tried to get out for walks.” (Interview 9)*.

Sleep deprivation and hormonal fluctuations were mentioned as drivers of more thoughts and concerns about physical changes and recovery. The initial time with the newborn sometimes deviates from expectations due to physical changes, seen in the subcategory *not as it was expected.* Concerns about unknown and surprising physical changes could influence the participants’ mental state, make them feel sad, and influence their ability to take care of the child. Some felt frustrated about this, whereas others said it just was to push through and that it did not influence their ability to be a mother.

*“Therefore*,* I mean*,* yeah*,* I had pain for quite a while and the worst part was that it was not what I had expected*,* so I felt frustrated. I had no information about what I could expect*,* so my expectation was that I would have felt normal much earlier*,* which I did not.” (Interview 7)*.

#### Recovery as an accomplishment

This category describes how recovery is seen as an accomplishment where the woman expressed feelings of being disappointed of her body but also proud and thankful to the body. Comparisons with others are made and perceived as a stressor. In the subcategory *disappointed with my body*, women expressed surprise at the prolonged recovery period after childbirth, which exceeded their expectations. The experienced need to always strive to be a good mother, care for their child, go for walks, and socialize with family and friends was experienced as a stressor for recovery. Disappointment arises from both their own and external pressures. An ambivalent relationship with today´s expectations of women´s postpartum recovery emerged: They expressed a desire for an active, healthy lifestyle without being part of the frenzy of getting in shape in record time.

*“I felt that I had to go for walks… and we lived in an apartment with 3 flights of stairs up and I had a dog*,* and it also had to go out and I was carrying and lifting and so on*,* yes*,* I think it was one of the causes that I did not heal” (Interview 10)*.

Conversely, when they are *proud and thankful to my body*, women express humility and pride in their bodies’ resilience and strength during childbirth and recovery. Despite the strain and sleep deprivation, they are grateful for their bodies’ endurance and ability to recover.

*After my childbirth*,* I experienced my body in a way that made me feel that my body was very amazing and powerful just because I had gone through childbirth… and that it had managed it. (Interview 1)*

The subcategory *comparing to others* reveals participants’ tendency to use the internet for comparison and to seek explanations for their recovery process. Gratitude for the fact that they got off “fairly well” contrasts with being frightened by others’ bad example stories or the experience that their own recovery takes more time than others’. Avoiding the internet was a strategy to not arouse insecurity or fear.

#### Sadness over losing my Prepregnant body

This category describes the experience of a changed body that causes sadness, as it is no longer the same as before. It is based on longing for the prepregnant body, which is often stronger and more active. Additionally, this feeling arises from not feeling seen or understood in their postpartum bodies. In the subcategory *longing for my prepregnant body*, participants expressed impatience at not immediately regaining their prepregnant bodies after childbirth. They anticipated the end of pregnancy as the moment when they would regain control over their bodies, only to find themselves navigating new and unfamiliar symptoms. The participants described how they experienced that they had lost some of themselves due to physical limitations.

*“It was a big adjustment*,* maybe I thought more about it during pregnancy*,* that I should adapt some exercises and such*,* but I could still do workouts and walks*,* but (afterwards) it took quite a long time before I felt I could handle walking again.” (Interview 6)*.

*Looking normal but feeling different* highlights the sense of injustice as attention shifts from the mother to the baby immediately after birth. The participants described feeling “used up”. Sadness is also built on the expression of being vulnerable and weak while already looking normal again. No one can see how they are feeling, which can lead to society’s misconception of how quickly women should recover after pregnancy.

*“There is not a lot of talk about how hard it is to recover after childbirth. I thought about that when I met some friends a week after the baby was born. None of those friends have children. Nobody thinks about…that you’re sitting there with your aching*,* stitched vagina.” (Interview 12)*.

#### Struggling with adjustment to physical changes

This category describes the challenges of adjusting to various pain conditions and bodily changes after pregnancy, including back pain, migraines, vaginal discomfort, abdominal pain, wrist pain, leakage, and bladder and bowel problems. In the subcategory *experiencing limitation*, it is shown how pain in different parts of the body made it difficult to care for their newborns during the early postpartum months. Support from family and friends was needed. Physical changes after pregnancy often affect the core of the body, which the participants experienced as challenging because it influences everything they do.

*It is okay now but not forever* describes how participants accepted transient changes in function and appearance, prioritizing basic functions such as bowel movements and carrying their child initially. Other activities, such as sexual function, running, or lifting heavy objects, were initially lower priorities but regained importance over time.

*“Right now*,* the focus is on so many other things now*,* so the sexual part is not like it was before pregnancy and childbirth. However*,* after a while you will want to find your way back to each other on that level as well… so I am a little bit worried that it will be uncomfortable” (Interview 5).*

The *process of accepting physical changes* varied among women, with acceptance viewed as a gradual process influenced by the disabling nature of the changes and associated fears and concerns. Some changes significantly impact daily life, leading to adjustments in routines or avoidance of triggering activities.

*“I can get symptoms that last*,* for example*,* until the next day*,* so I don´t truly dare to do it (the activity) right now.” (Interview 4)*.

The subcategory of *feeling powerless* reflects how women attempted to manage their problems through exercise and physiotherapy. When these efforts, such as pelvic floor muscle training, proved ineffective, they often felt powerless and uncertain about whether they were performing the exercises correctly. This sense of powerlessness was reinforced by the experience of not being seen or listened to by healthcare providers and by the perception that no professional assumed responsibility for addressing their concern:

*“I had a feeling of heaviness; I was in a lot of pain… well*,* it didn´t feel good. And then she (midwife) did the examination*,* and said*,* yes*,* everything looks good*,* the stitches have healed*,* so it looks good. I said*,* yes*,* but it does not feel good…but because she said*,* everything it looks fine*,* the stitches have healed and so on. Therefore… Yes*,* it seemed to be part of the normal*,* so*,* so I felt that I do not have to seek help for this.” (Interview 10)*.

#### Fear of not regaining my bodily functions

The unknown experience of physical changes and symptoms was aggravated by no or misleading information, causing fear that the body would not regain all functions or would develop problems later on. In the subcategory *fear caused by knowledge*, turning to the internet for information can exacerbate concerns due to conflicting or negative information. Family history, personal experiences, and misinformation from healthcare providers also contribute to anxiety. *Destroyed my body* showed how women feel abandoned with no or little information about experiences and thoughts they had due to pain, pelvic floor tears, or scars that did not heal. They feared irreversible damage and questioned whether they would ever fully recover.

*I gathered the courage to check how it looked down there when I had so much pain*,* and it was completely open. I became worried about the infections; I was afraid it would heal incorrectly. That it would give me future problems. (Interview 14)*

The fear of not regaining all bodily functions was compounded by the need to be physically strong to care for their children, as highlighted by *the need for a strong body to take care of my kids*. Having a baby (and toddlers) puts much strain on the body, they must be able to carry, lift and play several hours per day. They were afraid that a weak body could cause a chain reaction of more problems and pain, as they were *afraid of doing something wrong*. Women were scared of actively doing something wrong that could cause future problems. A weak pelvic floor was experienced as a “ticking time bomb” for prolapse. Guidance by health care givers was requested to answer these questions, but it was experienced that many health care givers did not know what was right nor wrong. The discrepancy between the women’s own experience and the health care givers’ assessments led to insecurity among the women.

*“I did not know where the limit was because the only thing that had been said or that I had read was to be cautious now with things that strain the pelvic floor. Not running was quite easy- considering that I do not normally run anyway. However*,* how much I could walk*,* for example*,* I did not know.” (Interview 2)*.

## Discussion

Women experience a range of activity limitations within the first 12 months after childbirth. While many difficulties are temporary and resolve within 3–6 months—such as challenges with brisk walking and exercising—others persist, including high-impact activities (e.g., running and jumping), childcare tasks (lifting and carrying), and, in some cases, basic functions (e.g., emptying the bowel and bladder) and intimacy. Pain frequently emerged as the primary cause of perceived limitations. Over the postpartum year, the underlying causes shifted: sensations of vaginal heaviness, weakness, instability, and uncertainty about readiness to exercise gradually decreased, whereas urinary incontinence became increasingly prominent as a barrier.

This pattern was also reflected in the qualitative findings. Women often reported being surprised and unprepared for physical changes during the first months, describing unfamiliar sensations and a need for greater understanding. Vaginal heaviness, in particular, was associated with concerns about the risk of “doing something wrong,” which may explain its role in limiting activity. While some participants trusted the body’s capacity for recovery, others described uncertainty, a strong desire to understand, difficulties adapting, and fears of not regaining full function—factors underlying why sensations of weakness and instability were especially limiting in the early months. Pain and incontinence can persist across the first year, as seen in both the quantitative and the qualitative analyses. Taken together, findings from both cohorts underscore the need for tailored support—providing reassurance and information about what is considered normal in the first 3–6 months and prioritizing targeted treatment for persistent symptoms such as pain and incontinence to avoid long-term limitations.

These findings highlight the need to address not only physical symptoms but also the psychological dimensions of postpartum recovery, as unexpected symptoms and uncertainty about the body’s responses can leave women feeling unprepared and vulnerable when receiving care and information postpartum. This aligns with theoretical perspectives that describe pregnancy and the postpartum period as a life transition characterized by insecurity and heightened vulnerability [27]. Although the concept of transition to motherhood has emphasized primarily the mother–child relationship and psychological adaptation[20], physical changes and recovery are integral to this transition. Physical changes can generate insecurity about what is normal, and women frequently express a need for information and support. At the same time, many were reluctant to burden the healthcare system, leaving their concerns largely invisible unless explicitly addressed. Similar findings have been reported elsewhere, highlighting the importance of acknowledging women’s concerns, listening to them, and treating persistent problems to prevent long-term symptoms such as urinary incontinence, pelvic girdle pain, and sexual or mental health concerns that can restrict physical activity and impair quality of life [28]. The concern that physical changes could affect future functionality, as seen in Cohort 2, has also been reported in a meta-synthesis of women’s postpartum care needs [29]. Importantly, most women want to know what is normal and what to expect—an observation consistent with studies on pelvic floor tears [17] and postpartum pain [30]. Our study further contributes by showing how unexpected and surprising these physical changes are for many women, which may explain why adaptation and fewer perceived limitations often require several months.

The need for social, professional, and informational support has been highlighted in several studies about pelvic girdle pain and pelvic floor tearing after pregnancy [31–34]. Conversely, women often postpone seeking help as they hope for natural recovery [30, 35]. An explanation could be that they are ashamed of their problems, do not want to be a burden for the health care system or do not know where to seek help, as seen in our study and another study [28]. A lack of trust in the competence of healthcare providers can be an additional problem, as also described elsewhere [16]. If women do not know where to seek help, they often gather large amounts of information online. Although the internet is an important information source for new mothers, it can also increase stress and concerns during the postpartum phase, as confirmed by another study [29].

Our study revealed that women want to feel seen and want to play an active role in recovery. To actively engage in their recovery, women need guidance on adapting activities that may trigger pain or leakage. The reported activity limitations in *Cohort 1* indicate that women mainly experience problems with recreational activities such as high-impact activities and exercising but also with walking and lifting/carrying during the first six months after childbirth. This finding is consistent with other studies, which revealed that 77% of mothers experienced pain-related interference with daily activities during the first two months after childbirth [36] and that approximately 5% encountered challenges with childcare activities within the first six months [37]. Pain and urinary incontinence are well-known causes of activity limitations[6, 38], whereas vaginal heaviness is less frequently reported in the literature. In the short term, enabling physical activity and exercising might be important not only for recovery from postpartum symptoms of urinary incontinence and pelvic girdle pain [22] but also for mental health [39]. Furthermore, the perceived inability to resume activities such as running can have long-term implications for women’s health, given the benefits of such activities, for example, for metabolic and cardiovascular diseases and bone health [40–42]. 

Physiotherapist-led interventions and/or guidance for self-management can address underlying causes of activity limitations, such as pain, weakness, or leakage [12, 43]. For example, pelvic floor muscle exercises are an effective treatments for urinary incontinence [44]. However, as our study demonstrated, women often feel insecure about whether they are performing these exercises correctly and experience powerlessness when the exercises do not alleviate their symptoms. Research has shown that more than half of women have difficulty activating their pelvic floor muscles correctly after childbirth—an issue that can be effectively addressed through feedback from a physiotherapist [45]. Both physical reassurance and verbal guidance, along with providing information, can play a crucial role in helping women build confidence in their body’s recovery, cope with persisting problems and empower them to actively adapt to changes. This concept is also exemplified in a postpartum health promotion model [46] and could be important for improving postpartum care.

### Strengths and limitations

This study benefits from data collection across two cohorts, offering both breadth and depth in understanding postpartum physical changes and activity limitations. However, this study has several limitations. The analysis of *Cohort 1 *was a secondary analysis, meaning that the original study design was not fully tailored to the specific research question. Consequently, women with major pelvic floor tears were excluded which might have limited insight into more severe symptoms and activity limitations. Several qualitative studies have addressed the experiences of, and perceived limitations caused by, these injuries[33, 47, 48], which are valuable in complementing the knowledge gained from this article. Another limitation is that we were unable to analyse which reported activity limitations were linked to specific causes, as participants first completed the PSFS and then listed causes in free text (see Appendix). It would have been methodologically inappropriate to assume a direct correspondence between the order of reported activities and causes.

Subjectivity in content analysis can be both a strength and a limitation. The quality of content analysis—either quantitative or qualitative—depends on the careful selection of participants as well as the experience and reflexivity of the researchers [49]. To enhance trustworthiness, several measures were employed to address credibility, dependability, confirmability, and transferability. Credibility was supported by recruiting participants with diverse experiences in Cohort 2 and from different parts of Region Västra Götaland, although women with lower educational levels were underrepresented, which may limit transferability. Only two women with lower educational levels agreed to participate in the interviews. While their experiences of physical changes and recovery were similar to those of the others, it is still questionable whether the results are transferable to other contexts or cultures [25]. Dependability was enhanced by continuous seven-month data collection, which minimized variations in the interviewer technique[25], with the risk that participants may have been influenced by current postpartum care trends or social media. Credibility was supported by conducting some of the interviews by phone in participants’ homes, which increased comfort and openness but limited the observation of facial expressions and spontaneous reactions [50]. Confirmability was addressed through reflection on researcher backgrounds: the first author, a physiotherapist specializing in postpartum care, conducted the analyses. Her clinical expertise might influence her objectivity toward participant experiences [51]. By staying closely aligned with the text during meaning unit condensation[49], she ensured that she told the participant´s story instead of her own experiences (examples in Table 3). All higher-level interpretations were discussed with a team of experienced qualitative researchers (GR, AG, MFO, and MEHL) to ensure rigor.

An additional limitation is that *Cohort 1 *participants completed an extensive questionnaire containing eight patient-reported outcome measures and background questions (other parts presented in previous publications)[21, 22, 52], with the PSFS as the final component. Response fatigue could have impacted response quality and completeness. Some participants might have chosen from the provided example causes for activity limitations instead of describing their own (see appendix). Moreover, there is a potential misunderstanding of the term “activities,” with respondents potentially overlooking issues related to bladder and bowel function, as well as sexual activity, considering them nonactivities. A study revealed that 38.3% of women experienced dyspareunia and that 6.2% experienced incomplete bowel evacuation one year after childbirth[53], which could indicate that the number of these activity limitations in our study was underestimated.

## Conclusion

This study, which combines data from two cohorts, revealed that women experience activity limitations during the first year post-partum, both due to symptoms such as pain and incontinence and due to uncertainty and unfamiliar bodily sensations. Many were unprepared for these physical changes and attempted to adapt without knowing what was appropriate. While some trusted their body’s ability to recover, others expressed a need for greater support.

Health care providers should offer timely information and guidance to help individuals navigate the postpartum transition. Addressing uncertainty about sensations such as weakness, instability, and vaginal heaviness—through reassurance, physical assessment, and tailored exercise—could help reduce early activity limitations. Uncertainty appeared most prominent in the early postpartum phase, whereas pain and incontinence persisted as limiting symptoms for around 30% of participants. Postpartum care may therefore need to be tailored, with reassurance and information early on and targeted treatment for persistent pain and incontinence later in recovery.

## Supplementary Information

Supplementary Material 1.

## Data Availability

The datasets from this study are not publicly available, as they consist of written and verbal quotes that could reveal the identities of individuals. However, the datasets are available from the corresponding author upon reasonable request.

## Abbreviations

BMI: Body mass index
PSFS: Patient-specific functional scale

## References

[1] Reimers C, et al. Change in pelvic organ support during pregnancy and the first year postpartum: a longitudinal study. BJOG Int J Obstet Gynaecol. 2016;123:821–9. 10.1111/1471-0528.13432 26113145

[2] Bø K, et al. Recovery of pelvic floor muscle strength and endurance 6 and 12 months postpartum in primiparous women—a prospective cohort study. Int Urogynecol J. 2022;33:3455–64. 36048249 10.1007/s00192-022-05334-y PMC9666345

[3] Sigurdardottir T, et al. Cross-sectional study of early postpartum pelvic floor dysfunction and related bother in primiparous women 6–10 weeks postpartum. Int Urogynecol J. 2021;32:1847–55. 33938963 10.1007/s00192-021-04813-y

[4] Palmieri S, et al. Prevalence and severity of pelvic floor disorders in pregnant and postpartum women. Int J Gynaecol Obstet. 2022;158:346–51. 34778951 10.1002/ijgo.14019

[5] Mota P, Pascoal AG, Carita AI, Bø K. Normal width of the inter-recti distance in pregnant and postpartum primiparous women. Musculoskelet Sci Pract. 2018;35:34–7. 29494833 10.1016/j.msksp.2018.02.004

[6] Gutke A, Lundberg M, Östgaard HC, Öberg B. Impact of postpartum lumbopelvic pain on disability, pain intensity, health-related quality of life, activity level, kinesiophobia, and depressive symptoms. Eur Spine J. 2011;20:440–8. 20593205 10.1007/s00586-010-1487-6 PMC3048223

[7] Almalik MM. Understanding maternal postpartum needs: a descriptive survey of current maternal health services. J Clin Nurs. 2017;26:4654–63. 28329433 10.1111/jocn.13812

[8] Tully KP, Stuebe AM, Verbiest SB. The fourth trimester: a critical transition period with unmet maternal health needs. Am J Obstet Gynecol. 2017;217:37–41. 28390671 10.1016/j.ajog.2017.03.032

[9] McCarter D, MacLeod CE. What do women want? Looking beyond patient satisfaction. Nurs Womens Health. 2019;23:478–84. 31672402 10.1016/j.nwh.2019.09.002 PMC6931278

[10] National Board of Health and Welfare (Sweden). Pregnant and Postpartum Women’s Situation and Needs (Gravida Och Nyförlösta Kvinnors Situation Och Behov). https://www.socialstyrelsen.se/globalassets/sharepoint-dokument/artikelkatalog/ovrigt/2019-11-6436.pdf (2019).

[11] Higgs K, Refshauge Elizabeth J. Portrait of the physiotherapy profession. J Interprof Care. 2001;15:79–89. 11705073 10.1080/13561820020022891

[12] Critchley CJC. Physical therapy is an important component of postpartum care in the fourth trimester. Phys Ther. 2022;102:pzac021. 35225339 10.1093/ptj/pzac021

[13] Srisopa P, Lucas R. Women’s experience of pelvic girdle pain after childbirth: a meta-synthesis. J Midwifery Womens Health. 2021;66:240–8. 33314586 10.1111/jmwh.13167

[14] Christopher SM, Cook CE, Snodgrass SJ. What are the biopsychosocial risk factors associated with pain in postpartum runners? Development of a clinical decision tool. PLoS ONE. 2021;16:e0255383. 34383792 10.1371/journal.pone.0255383 PMC8360599

[15] Olsson A, Lundqvist M, Faxelid E, Nissen E. Women’s thoughts about sexual life after childbirth: focus group discussions with women after childbirth. Scand J Caring Sci. 2005;19:381–7. 16324063 10.1111/j.1471-6712.2005.00357.x

[16] Henshaw EJ, et al. Trying to figure out if you’re doing things right, and where to get the info: parents recall information and support needed during the first 6 weeks postpartum. Matern Child Health J. 2018;22:1668–75. 29978309 10.1007/s10995-018-2565-3

[17] Daremark C, Andréasson L, Gutke A, Fagevik Olsén M. Women’s experiences of the injury, recovery and desire for rehabilitation after a second-degree vaginal tear—a qualitative study. Int Urogynecol J. 2022;33:1521–7. 34370062 10.1007/s00192-021-04951-3 PMC9206620

[18] Martin A, Horowitz C, Balbierz A, Howell EA. Views of women and clinicians on postpartum preparation and recovery. Matern Child Health J. 2014;18:707–13. 23775250 10.1007/s10995-013-1297-7 PMC4304667

[19] Schytt E, Lindmark G, Waldenström U. Physical symptoms after childbirth: prevalence and associations with self-rated health. BJOG: An International Journal of Obstetrics & Gynaecology. 2005;112:210–7. 15663586 10.1111/j.1471-0528.2004.00319.x

[20] Barimani M, Vikström A, Rosander M, Forslund Frykedal K, Berlin A. Facilitating and inhibiting factors in transition to parenthood – ways in which health professionals can support parents. Scand J Caring Sci. 2017;31:537–46. 28144992 10.1111/scs.12367

[21] Vesting S, et al. Can clinical postpartum muscle assessment help predict the severity of postpartum pelvic girdle pain? A prospective cohort study. Phys Ther. 2022;103:pzac152. 36326139 10.1093/ptj/pzac152 PMC10071582

[22] Vesting S, Gutke A, Fagevik Olsén M, Rembeck G, Larsson MEH. The impact of exercising on pelvic symptom Severity, pelvic floor muscle Strength, and diastasis recti abdominis after pregnancy: A longitudinal prospective cohort study. Phys Ther. 2024;104:pzad171. 38109793 10.1093/ptj/pzad171 PMC11021861

[23] Stratford P. Assessing disability and change on individual patients: a report of a patient specific measure. Physiother Can. 1995;47:258–63.

[24] Krippendorff K. Content analysis: an introduction to its methodology. Thousand Oaks, CA: Sage; 2004.

[25] Graneheim UH, Lundman B. Qualitative content analysis in nursing research: concepts, procedures and measures to achieve trustworthiness. Nurse Educ Today. 2004;24:105–12. 14769454 10.1016/j.nedt.2003.10.001

[26] Malterud K, Siersma VD, Guassora AD. Sample size in qualitative interview studies: guided by information power. Qual Health Res. 2016;26:1753–60. 26613970 10.1177/1049732315617444

[27] Meleis AI, Sawyer LM, Im E-O, Messias H, D. K., Schumacher K. Experiencing transitions: an emerging Middle-Range theory. Adv Nurs Sci. 2000;23:12–28. 10.1097/00012272-200009000-00006 10970036

[28] Daly D, et al. Trajectories of postpartum recovery: what is known and not known. Clin Obstet Gynecol. 2022;65:594–610. 35797600 10.1097/GRF.0000000000000726

[29] Finlayson K, Crossland N, Bonet M, Downe S. What matters to women in the postnatal period: a meta-synthesis of qualitative studies. PLoS ONE. 2020;15:e0231415. 32320424 10.1371/journal.pone.0231415 PMC7176084

[30] Molin B, Zwedberg S, Berger A-K, Sand A, Georgsson S. Disempowering women—a mixed methods study exploring informational support about pain persisting after childbirth and its consequences. BMC Pregnancy Childbirth. 2022;22:510. 35739466 10.1186/s12884-022-04841-6 PMC9229078

[31] O’Reilly R, Peters K, Beale B, Jackson D. Women’s experiences of recovery from childbirth: focus on pelvis problems that extend beyond the puerperium. J Clin Nurs. 2009;18:2013–9. 19638059 10.1111/j.1365-2702.2008.02755.x

[32] Crookall R, Fowler G, Wood C, Slade P. A systematic mixed studies review of women’s experiences of perineal trauma sustained during childbirth. J Adv Nurs. 2018;74:2038–52. 10.1111/jan.13724 29791012

[33] Johannesson E, Sjöberg Å-L, Segerbrand N, Fagevik Olsén M, Gutke A. Women’s experiences of obstetric anal sphincter injury and physical therapy interventions - a qualitative study. Braz J Phys Ther. 2022;26:100397. 35364345 10.1016/j.bjpt.2022.100397 PMC8971829

[34] Wuytack F, Curtis E, Begley C. Experiences of first-time mothers with persistent pelvic girdle pain after childbirth: descriptive qualitative study. Phys Ther. 2015;95:1354–64. 25929535 10.2522/ptj.20150088

[35] Buurman MBR, Lagro-Janssen ALM. Women’s perception of postpartum pelvic floor dysfunction and their help‐seeking behaviour: a qualitative interview study. Scand J Caring Sci. 2013;27:406–13. 22924517 10.1111/j.1471-6712.2012.01044.x

[36] Declercq ER, Sakala C, Corry MP, Applebaum S, Herrlich A. Major survey findings of listening to Mothers^SM^ III: pregnancy and birth: report of the third national U.S. survey of women’s childbearing experiences. J Perinat Educ. 2014;23:9–16. 24453463 10.1891/1058-1243.23.1.9 PMC3894594

[37] Tulman L, Fawcett J, Groblewski L, Silverman L. Changes Funct Status after Childbirth: Nurs Res. 1990;39:70–5. 2315069

[38] Åhlund S, Rothstein E, Rådestad I, Zwedberg S, Lindgren H. Urinary incontinence after uncomplicated spontaneous vaginal birth in primiparous women during the first year after birth. Int Urogynecol J. 2020;31:1409–16. 31139858 10.1007/s00192-019-03975-0 PMC7306031

[39] Badon SE, Iturralde E, Nkemere L, Nance N, Avalos LA. Perceived barriers and motivators for physical activity in women with perinatal depression. J Phys Act Health. 2021;18:801–10. 33984835 10.1123/jpah.2020-0743 PMC9851467

[40] Lavie CJ, et al. Effects of running on chronic diseases and cardiovascular and all-cause mortality. Mayo Clin Proc. 2015;90:1541–52. 26362561 10.1016/j.mayocp.2015.08.001

[41] Pedisic Z, et al. Is running associated with a lower risk of all-cause, cardiovascular and cancer mortality, and is the more the better? A systematic review and meta-analysis. Br J Sports Med. 2020;54:898–905. 31685526 10.1136/bjsports-2018-100493

[42] Vainionpää A, et al. Intensity of exercise is associated with bone density change in premenopausal women. Osteoporos Int. 2006;17:455–63. 16404492 10.1007/s00198-005-0005-x

[43] Simonds AH, Abraham K, Spitznagle T. Clinical practice guidelines for pelvic girdle pain in the postpartum population. J Womens Health Phys Ther. 2022;46:E1–38.

[44] Mørkved S, Bø K. Effect of pelvic floor muscle training during pregnancy and after childbirth on prevention and treatment of urinary incontinence: a systematic review. Br J Sports Med. 2014;48:299–310. 23365417 10.1136/bjsports-2012-091758

[45] Neels H, De Wachter S, Wyndaele J-J, Van Aggelpoel T, Vermandel A. Common errors made in attempt to contract the pelvic floor muscles in women early after delivery: a prospective observational study. Eur J Obstet Gynecol Reprod Biol. 2018;220:113–7. 29202394 10.1016/j.ejogrb.2017.11.019

[46] Fahey JO, Shenassa E. Understanding and meeting the needs of women in the postpartum period: the perinatal maternal health promotion model. J Midwifery Womens Health. 2013;58:613–21. 24320095 10.1111/jmwh.12139

[47] Lindqvist M, Lindberg I, Nilsson M, Uustal E, Persson M. Struggling to settle with a damaged body’ - a Swedish qualitative study of women’s experiences one year after obstetric anal sphincter muscle injury (OASIS) at childbirth. Sex Reprod Healthc Off J Swed Assoc Midwives. 2019;19:36–41. 10.1016/j.srhc.2018.11.002 30928133

[48] Darmody E, Bradshaw C, Atkinson S. Women’s experience of obstetric anal sphincter injury following childbirth: an integrated review. Midwifery. 2020;91:102820. 32861872 10.1016/j.midw.2020.102820

[49] Graneheim UH, Lindgren B-M, Lundman B. Methodological challenges in qualitative content analysis: a discussion paper. Nurse Educ Today. 2017;56:29–34. 28651100 10.1016/j.nedt.2017.06.002

[50] Novick G. Is there a bias against telephone interviews in qualitative research? Res Nurs Health. 2008;31:391–8. 18203128 10.1002/nur.20259 PMC3238794

[51] Korstjens I, Moser A, Series. Practical guidance to qualitative research. Part 4: trustworthiness and publishing. Eur J Gen Pract. 2018;24:120–4. 29202616 10.1080/13814788.2017.1375092 PMC8816392

[52] Cristóvão S, Asplén E, Borssén J, Larsson MEH, Vesting S. Pelvic floor muscle strength and bothersome urinary incontinence after pregnancy: a cohort study. Int Urogynecol J. 2025. 10.1007/s00192-025-06085-2. 40080111 10.1007/s00192-025-06085-2 PMC12464028

[53] Huber M, Malers E, Tunón K. Pelvic floor dysfunction one year after first childbirth in relation to perineal tear severity. Sci Rep. 2021;11:12560. 34131194 10.1038/s41598-021-91799-8 PMC8206367

