# Supplementary material for: Surprised by the transition to an unknown body: quantitative and qualitative aspects of activity limitations and physical changes during the first year post-partum
Source: BMC Pregnancy Childbirth. 2025 Oct 2;25:1002. doi: 10.1186/s12884-025-08224-5 (PMC12490155; doi:10.1186/s12884-025-08224-5)
Supplement: Supplementary file 1 — Supplementary Material 1. [file 12884_2025_8224_MOESM1_ESM.pdf]

1. PSFS- Patient-specific functional scale

We would like to ask you to describe up to three important activities that you find difficult or are unable to perform as a result of giving birth (due to physical problems such as back/pelvic pain, urinary incontinence, or vaginal heaviness).

Which activities do you find difficult to perform?

Please enter the number on the scale that best reflects how difficult each activity is for you.

(If you can't think of any activities you struggle with, feel free to leave the following fields blank.)

Activity 1: ____

0	1	2	3	4	5	6	7	8	9	10
☐	☐	☐	☐	☐	☐	☐	☐	☐	☐	☐

Unable to perform activity

Able to perform activity without difficulty or at the same level as before pregnancy

Activity 2: ____

0	1	2	3	4	5	6	7	8	9	10
☐	☐	☐	☐	☐	☐	☐	☐	☐	☐	☐

Unable to perform activity

Able to perform activity without difficulty or at the same level as before pregnancy

Activity 3: ____

0	1	2	3	4	5	6	7	8	9	10
☐	☐	☐	☐	☐	☐	☐	☐	☐	☐	☐

Unable to perform activity

Able to perform activity without difficulty or at the same level as before pregnancy

We would like to ask you to write the cause of why you are unable to perform the activity or activities:

____

____

2. Interview guide

- *How did you experience your body after pregnancy?*
- *Is there any difference compared to before?*
- *How do you feel about this? Do you have positive or negative feelings?*
- *How recovered do you feel now?*
- *How does this affect for in your role as a mother?*
- *How do you experience your body when you move?*
- *How does this affect you as a person?*
- *Is there anything that limits you?*
- *How did you experience the help you received; would you have wished for something else?*

The questions were deepened by further asking of "how do you mean this?", "how did you experience this", "can you explain for me.."

Table 1. The analysis process from examples of condensed meaning units to codes, subcategories and categories

Categories	Subcategories	Codes	Examples of condensed meaning units
An unfamiliar bodily sensation	Helpful to be prepared	Having experiences	Knowing from the first child that vaginal heaviness will
		Never felt this before	It is hard to now as you never have felt
		Being prepared	Not sure if pre-natal information about postpartum would help
	Cannot do it alone, help me	Support of family and friends	Needed my mother/sister to talk
		Support of health care	Helpful to find a physiotherapist
		Needing reassurance	Want a check-up for reassurance of bodily changes
	Just want to know/understand	Needing information	Started to read about it online
		Finding own explanations online	I have low back pain due to my abdominal separation
		Asking for the new normal	I think everything I feel is normal but I am a little bit unsure about it
	Active role in recovery	Should I do something	Want to know if this is the new normal or if I should do something
		Needing the right tools	Not sure if I am doing the pelvic floor exercises right
		Needing the right help	Not sure if I should rest or do more to treat my vaginal heaviness
Trusting the body´s ability to recover	Feeling good now	Feeling recovered	My body feels recovered
		Confident about the body´s recovery	I feel confident in testing my body´s limitations
	Changed but acceptable	The body´s function is important	As long as I have a functional body, I can accept some pain
	Getting better and better	Needing rest and time for recovery	I have some pelvic girdle pain sometimes but it is always getting better of resting
		Experiencing small successes	I can go longer and longer again
	Having control	Will get strong again	My exercises will help me to get strong again
		Being responsible	My recovery is my own responsibility
Sadness of losing my pre-pregnant body	Longing for my pre-pregnant body	Impatience to live a normal life again	Do not want that my body still affects my everyday life
		Focus shifts from mother to baby	Everything is about the baby now, but I need care too.
		Cannot be as active as I want to be	Feeling jealous of my partner who can run an activity we used to do together
	Looking normal but feeling different	Feeling/being teared	I look normal and nobody knows how teared I feel
		Feeling misunderstood	It looks fine but I feel not fine
Challenged by the overload of being mother to a new-born	Feeling limited in energy	Hard to take care about my own recovery	Inner conflict about the need of rest and activity
		Stressed by the need to take care of myself	There is not enough energy to take care of the body right now
		Need of help	Needed all help I could get
	Overwhelmed of the first weeks	Adding up with other problems	Pain increased because of other problems like fever and mastitis
		Pain is secondary due to other problems	No time to think about bodily changes in the first weeks

		Getting worse by sleep deprivation	Feeling more anxious than necessary due to sleep deprivation, hormones mm
	Not as it was expected	More problems than expected	Could not go for longer walks during the first month
		Wrong expectations	Want to feel good during the first time with the baby
Struggling with the adjustment to physical changes	Experiencing limitations	Experiencing pain/leakage	Difficulties to take care of my child due to pain
		Threatening basic function (bowel and bladder)	Feeling discomfort and instability when emptying the bowel
		Affecting the core	Pain in the central parts of the body affects everything
	It´s okay but not forever	Recovery takes time	The body is weakened after childbirth
		Shifting priorities	Sexual activities are less important right now but will be more important again in the future
	A process of accepting	Need to accept changes in appearance	Struggling with accepting that the body looks different after having a baby
		Need to accept changes in function	Tearing and pain affect my functions
	Need of adapting	Adapting my everyday life	I am adapting my daily routines to leakage
		Avoiding activities	Avoid certain activities because of pain after the activity
	Feeling powerless	Cannot influence my recovery	Pelvic floor exercises did not help against my urgency incontinence
		Nobody is helping me	Feels like nobody is responsible for me
Recovery as an accomplishment	Disappointed of my body	Pressured by others	Nowadays people expect that you do and exercise so much after childbirth
		Pressured by myself	Disappointed that my body recovered so slowly
		Striving after to be a good mother	Could not go for walks with the stroller in the first months
	Proud and thankful to the body	Impressed by the body	Impressed of my body's ability to recover so quickly
		Fascinated by the body`s ability to be strong	Fascinated that the body can do such a thing and then recover relatively quickly
		Humbled of the body´s abilities	Thought that appearance would be more important, instead a feeling of gratitude to the body
	Comparing to others	Being the only one	Thinking that I was the only one who was having a hard time because of the internet
		Could be worse	Social media posts about pelvic floor tearing has made me feel like I'm lucky
		Terrified by stories of others	Reading horror stories about tearing scared med
Fear of not regaining my bodily functions	Fear caused by knowledge	Reinforced fear by the internet	Information from the internet made me think the feeling of heaviness was a prolapse
		Having risk factors in family history	Having relatives who needed surgery for pelvic floor problems scared me
	Destroyed my body	Unchangeable physical changes	Scared that my body will be like this forever
		Scared to be teared	Checked the tear with a mirror and was wondering how this can heal
	Needing a strong body to take care of my kids	Need a strong core	Life with small children demands a strong body
		Wondering what´s next	Am I able to have more children and an active life with these physical changes?
	Scared to do something wrong	Might be dangerous	Don´t dare to run/exercise, it could cause problems later on
		Feeling unstable	Vaginal heaviness can be a warning sign for prolapse
